# Three-dimensional resonating metamaterials for low-frequency vibration attenuation

**DOI:** 10.1038/s41598-019-47644-0

**Published:** 2019-08-08

**Authors:** W. Elmadih, D. Chronopoulos, W. P. Syam, I. Maskery, H. Meng, R. K. Leach

**Affiliations:** 1 Manufacturing Metrology Team, Faculty of Engineering, University of Nottingham, Nottingham, NG8 1BB UK; 2 Institute for Aerospace Technology & Composites Group, Faculty of Engineering, University of Nottingham, Nottingham, NG8 1BB UK; 3 Centre for Additive Manufacturing, Faculty of Engineering, University of Nottingham, Nottingham, NG8 1BB UK

**Keywords:** Materials science, Condensed-matter physics, Engineering

## Abstract

Recent advances in additive manufacturing have enabled fabrication of phononic crystals and metamaterials which exhibit spectral gaps, or stopbands, in which the propagation of elastic waves is prohibited by Bragg scattering or local resonance effects. Due to the high level of design freedom available to additive manufacturing, the propagation properties of the elastic waves in metamaterials are tunable through design of the periodic cell. In this paper, we outline a new design approach for metamaterials incorporating internal resonators, and provide numerical and experimental evidence that the stopband exists over the irreducible Brillouin zone of the unit cell of the metamaterial (i.e. is a three-dimensional stopband). The targeted stopband covers a much lower frequency range than what can be realised through Bragg scattering alone. Metamaterials have the ability to provide (a) lower frequency stopbands than Bragg-type phononic crystals within the same design volume, and/or (b) comparable stopband frequencies with reduced unit cell dimensions. We also demonstrate that the stopband frequency range of the metamaterial can be tuned through modification of the metamaterial design. Applications for such metamaterials include aerospace and transport components, as well as precision engineering components such as vibration-suppressing platforms, supports for rotary components, machine tool mounts and metrology frames.

## Introduction

Phononic crystals (PCs) are engineered materials designed to control elastic wave propagation. PCs generally rely on high impedance mismatches within their structural periodicity to form Bragg-type stopbands that exist due to the destructive interference between transmitted and reflected waves. The presence of destructive interference prevents specific wave types from propagating. Kushwaha *et al*.^1^ presented the first comprehensive calculation of acoustic bands in a structure of periodic solids embedded in an elastic background. James *et al*.^2^ used a periodic array of polymer plates submerged in water and provided experimental realisation of one-dimensional (1D) and two-dimensional (2D) PCs. Montero de Espinosa *et al*.^3^ used aluminium alloy plates with cylindrical holes filled with mercury to generate 2D ultrasonic stopbands. Tanaka *et al*.^4^ studied the homogeneity of PCs in the perpendicular direction to the direction of propogation, and classified PCs into bulk PCs and slab PCs. Research on the design, manufacturing and testing of PCs has mainly focused on 1D and 2D PCs^5–15^, although recently, the research has been extended to include 3D PCs^16–24^. Lucklum *et al*.^25^ discussed the manufacturing challenges of 3D PCs and showed that additive manufacturing (AM) has the fabrication capabilities required for the realisation of geometrically complex 3D PCs^26–29^. There are a wide variety of AM technologies that may be used to manufacture PC materials, such as laser powder bed fusion (LPBF), photo-polymerization, stereolithography and inkjet printing^30–33^. Although differing in the manufacturing resolution (the thickness of the build layer), materials, design constraints and cost, these AM technologies create 3D parts from a CAD model. The creation of the 3D parts is usually carried out layer by layer, and the thickness of the deposited layers, as well as the effects of post-processing, determine the geometrical quality of the created 3D parts^34,35^.

Despite the benefits of the recent ability to manufacture PCs with AM, their effectiveness at low-frequencies is limited due to the dependency of the resulting stopbands on Bragg scattering. Bragg scattering occurs due to destructive interference of the propagating waves with the in-phase reflected waves, which occurs when the wavelengths of the reflected and propagating waves are similar. The reflection occurs due to the difference in the impedance (e.g. local density) of the PC. For the in-phase reflection to occur, the Bragg law has to be satisfied^36^, which is highly dependent on the cell size of the PC. Bragg scattering starts to occur when the wavelength is approximately equal to twice the cell size of the PC^36^; around a normalised frequency (the quotient of cell size and wavelength) of 0.5. Thus, there is a limiting dependency on the size of the unit cell of the PCs to form stopbands by Bragg scattering. As a result of this dependency, unrealistic cell sizes need to be employed to satisfy the Bragg law at low-frequencies.

It is possible to form stopbands below the lowest Bragg limit using metamaterials with periodically arranged local resonators. The stopbands in these metamaterials are formed by absorbing wave energy around the resonant frequency^37–44^. The benefits of resonator-based metamaterials include increased design freedom and the flexibility to obtain stopbands in structures of higher periodicity within a fixed design volume compared to conventional PCs. Thus, resonator-based metamaterials provide better-defined stopbands. Research on locally resonant metamaterials includes the work of Liu *et al*.^44^, who first developed a metamaterial using solid cores and silicone rubber coatings. The periodically coated spheres of Liu *et al*. exhibited negative dynamic mass, as well as stopbands at low frequencies. Numerous locally resonant metamaterials have been proposed. An example by Fang *et al*.^45^ showed arrays of Helmholtz resonators with negative dynamic bulk modulus. Qureshi *et al*.^46^ numerically investigated the existence of stopbands in cantilever-in-mass metamaterials. Lucklum *et al*.^21^ and D’Alessandro *et al*.^47^ independently verified the existence of stopbands in ball-rod metamaterials. Zhang *et al*.^48^ presented results of a beam metamaterial with local resonance stopbands. Bilal *et al*.^49^ reported on the concept of combining local resonance with Bragg scattering to form *trampoline metamaterial* with subwavelength stopbands. Matlack *et al*.^50^ developed a multimaterial structure that has wide stopbands using similar concept to that of Bilal *et al*.^49^. Most of the above work, regarding both PCs and metamaterials, has employed analytical techniques to model and optimise the suggested unit cells. Because analytical techniques can only model simple designs, the potential for exploring the elastic capabilities of complex metamaterial designs has been limited.

**Figure 1:**
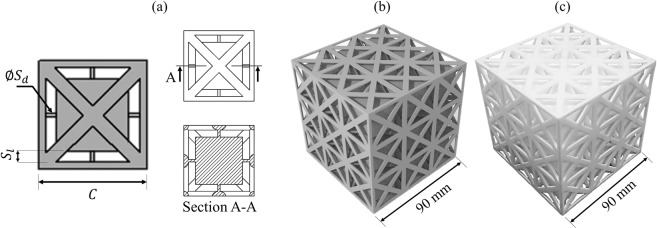
The design of the resonating metamaterial: (**a**) Schema of the single unit cell of the metamaterial as modelled in CAD, the labels show the strut diameter (*S*_*d*_), strut length (*S*_*l*_), and cell size (*C*), and photograph of the 3 × 3 × 3 metamaterial as (**b**) digitally rendered, and (**c**) manufactured with LPBF.

**Figure 2:**
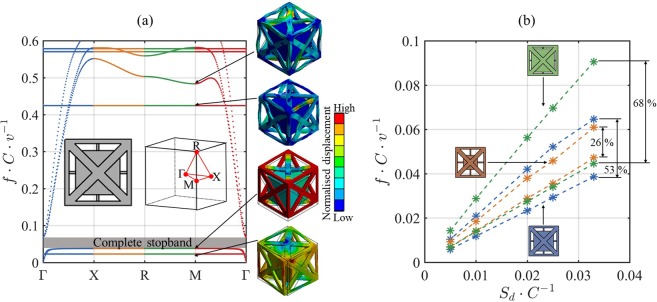
Wave propagation properties of the internally resonating metamaterial: (**a**) Dispersion curves for the metamaterial with *S*_*d*_*/C* and *S*_*l*_*/C* values of 0.033 and 0.1, respectively, with eigenmodes at selection of high symmetry points, and (**b**) start and end frequencies of the complete stopbands of metamaterials of different *S*_*d*_*/C* values with the struts connected to resonators of large-size (green), mid-size (blue), and small-size (orange). The indicated percentages show the relative gap to mid-gap percentage. All frequencies (*f*) are normalised to the longitudinal wave speed in the medium *v* and the unit cell size *C*.

**Figure 3:**
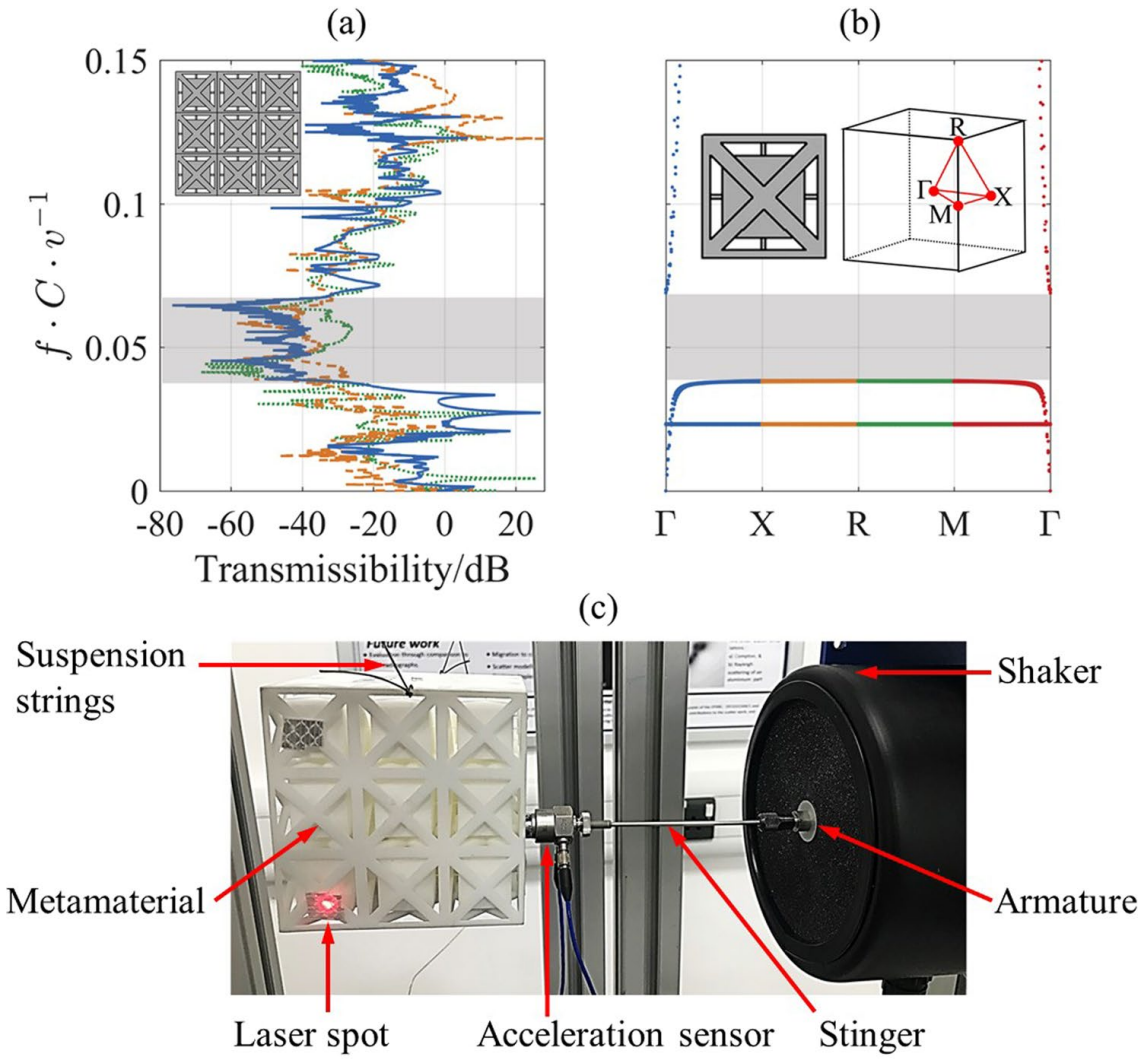
Experimental results acquired for the resonating metamaterial: (**a**) Transmissibility of the 3 × 3 × 3 metamaterial in the *x*- longitudinal direction (solid line), *y*- transverse direction (dotted line), and *z*- transverse direction (dashed line) vis-à-vis the corresponding stopband as illustrated by the dispersion curves of the infinite metamaterial shown in (**b**), and (**c**) representative photograph of the experimental setup. The shaded areas show the identified stopbands.

We hereby report on 3D metamaterial comprising internal resonators, designed for targeting maximum elastic wave attenuation below a normalised frequency of 0.1. This normalised frequency limit, chosen arbitrarily, is four times lower than the lowest theoretical limit allowed for Bragg scattering stopbands. Due to its high normalised stopband frequencies, a PC relies heavily on increasing the cell size to reduce the absolute stopband frequency. The low normalised stopband frequencies of metamaterials allow for vibration attenuation at low absolute frequencies using much more practical unit cell sizes (i.e. of more suitable dimensions for AM and applications). A novel approach for tuning and designing the unit cell of the metamaterial is presented. The computation scheme of the wave dispersion curves uses finite element (FE) modelling. In comparison to finite difference time domain (FDTD) modelling which suffers from stair-casing effects^51^, and plain wave expansion (PWE) modelling which is limited to structures of low impedance mismatch^52^, FE modelling guarantees an accurate description of the wave dynamics within the 3D metamaterial. LPBF is employed for fabrication of the metamaterial, which is experimentally tested for verification of the numerical predictions. The fundamental unit cell of the metamaterial is shown in Figure 1, and is periodically tessellated in 3D to allow a local resonance effect. The 3D wave propagation and the complete stopbands of the metamaterial are presented in Figure 2. The experimental response of the manufactured metamaterial is shown in Figure 3. Details of the computation, manufacturing and experimental methods are provided in the subsequent sections.

## Results and Discussion

The unit cell of the metamaterial featured in this work is shown in Figure 1. The design is a cubic unit cell with face-centered struts (FCC), and reinforcement struts in the *x*-, *y*- and *z*- directions (FCC_xyz_). FCC lattices generally have good compressive strength^53^, in comparison to body-centred cubic lattices (BCC). Thus, the FCC_xyz_ lattice is used as the host for the internal resonance mechanism of the metamaterial. The internal resonance mechanism consists of six struts; each connects one side of a cubic mass to the inner walls of the FCC_xyz_ unit cell. Increasing the strut diameter *S*_*d*_ would increase the stiffness of the resonator, while increasing the strut length *S*_*l*_ would alter its volume fraction, which will have an impact on the stopband frequencies and the total mass.

Modelling of the elastic wave propagation in the metamaterials was carried out in 3D using the scheme described in the Methods Section. The modelling used sufficient tetrahedral elements, such that the frequency of the first vibration mode converged with the FE mesh density (approximately 6000 nodes per unit cell). The elements of the converged mesh used three degrees of freedom (DOF) per node with adaptive mesh size to sufficiently model narrow regions in the metamaterials^54^. To mathematically model the elastic wave propagation, the contours of the irreducible Brillouin zone (IBZ) of the unit cells of the metamaterials were scanned. Several characteristic points exist within the contours of the IBZ including Γ(0,0,0), X(π/*C*,0,0), M(*π/C*,*π/C*,0), and R(*π/C*,*π/C*,*π/C*), where *C* is the unit cell size (also referred to as *α* or *L* in other literature^50,55,56^). The scan of the IBZ was carried out using a total of 360 combinations of wavenumbers (90 combinations for each wave propagation direction). The corresponding dispersion properties along the path Γ–X–R–M–Γ of the IBZ were predicted and the complete stopbands were identified. The dispersion curves of a metamaterial unit cell with *S*_*d*_/*C* and *S*_*l*_/*C* values of 0.033 and 0.1, respectively, are presented in Figure 2a. It was observed that the metamaterial exhibits a stopband below a normalised frequency of 0.1. The stopband spans a normalised frequency range of 0.028, starting from 0.039 to 0.067, and is formed by an internal resonance that cuts the first three acoustic wavebands (wavebands cutting-on at zero frequency) and splits them into two branches (i.e. top and bottom acoustic branches).

The dispersion curves of multiple metamaterials of different values of *S*_*d*_/*C* and *S*_*l*_/*C* were predicted. The considered *S*_*d*_/*C* values were 0.005, 0.01, 0.02, 0.025 and 0.033, and the considered *S*_*l*_/*C* values were 0.05 (large-size resonator), 0.1 (mid-size resonator) and 0.2 (small-size resonator). Figure 2b presents the stopbands for each of the considered metamaterials to show the impact of the design of the internal resonators on forming complete 3D stopbands. The relative gap to mid-gap percentages of selection of the presented stopbands (width of the stopband divided by its central frequency) are highlighted in Figure 2b. The large-size resonator showed the largest relative gap to mid-gap percentage of 68%. The cut-on frequency of the top acoustic branches (i.e. the stopband end frequency) increased with the increase in the diameter of the struts, and with the increase in the size of the resonator. The stopbands of all the considered unit cell designs were below a normalised frequency of 0.1, as can be seen in Figure 2b. The stopbands of the large-size resonator had wider stopbands than that of the mid-size resonator. The average stopband width in the large-size resonator was calculated to be wider by 63%, and 236% than that of mid-size and small-size resonators, respectively. The mean frequency of the stopband showed a change of 2.4% with the change in the resonator size. The results shown in Figure 2b can be used as a means of tuning the stopbands of the metamaterial for a specific application.

For verification of the complete stopband in the proposed metamaterial, LPBF was used to manufacture a 3D structure of finite periodicity. Details about the LPBF process can be found in the Methods Section. The geometrical dimensions and periodicity of the metamaterial were selected to be suitable for the LPBF process. The manufactured metamaterial, presented in Figure 1c, had a unit cell size of 30 mm and a 3D periodicity of three. The *S*_*d*_/*C* and *S*_*l*_/*C* values were selected to provide the lowest stopband start frequency, when referenced to the stopband start frequencies presented in Figure 2b while considering the lowest manufacturable feature size with LPBF^57^ (See Methods Section); this meant that the *S*_*d*_/*C* and *S*_*l*_/*C* values had to be 0.033 and 0.1, respectively. The 3D transmissibility of the metamaterial was obtained experimentally and is presented in Figure 3a. The longitudinal transmissibility had a value of 0 dB near the normalised frequency of zero, which indicates complete transmission of the excitation waves. At the vibration resonances, the longitudinal transmissibility was greater than 0 dB and reached 28 dB, which indicates high amplification of the excitation waves. Within the stopband, the longitudinal transmissibility reached −77 dB. The effect of lattice periodicity on the transmissibility within the stopband can be seen elsewhere^12,58^. For this investigation, considering the manufacturable feature size of LPBF (See Methods Section), we have chosen 3 × 3 × 3 as a reasonable example. The results showed that the metamaterial in this work has double the transmissibility reduction experimentally reported by Croënne *et al*.^12^ for their 3D PC which had double the spatial periodicity used in this work.

The 3D elastic wave propagation in the internally resonating metamaterials was modelled using a hybrid scheme. The scheme uses the FE method combined with infinite periodicity assumptions. It was shown that the metamaterials exhibit complete stopbands far below the lowest frequency limit of Bragg-type stopbands, which exist in traditional PCs. A metamaterial of finite periodicity was manufactured using LPBF. An experimental setup was assembled, comprising a broadband vibration shaker, a laser vibrometer, and dedicated signal generation and acquisition units. The experimental setup was used to test the 3D vibration transmissibility of the manufactured metamaterial. It was shown that the metamaterial could attenuate the vibration waves within the stopband range. The experimental results showed that, within the stopband, the longitudinal transmissibility of vibration waves in the metamaterial reached −77 dB. Tuning of the stopband can be achieved by adjusting the size of the resonator and the diameter of the struts to suit the requirements of various applications. For this particular metamaterial, the stopband was from 1.63 kHz to 2.8 kHz with a unit cell size of 30 mm. Unit cells of suitable dimensions for AM and applications, and higher periodicity within a certain design volume, in comparison to PCs, can be employed to obtain low absolute frequency stopbands; resulting in higher attenuation within the stopbands.

## Methods

### Modelling of elastic wave propagation using a hybrid wave and finite element scheme

**Figure 4:**
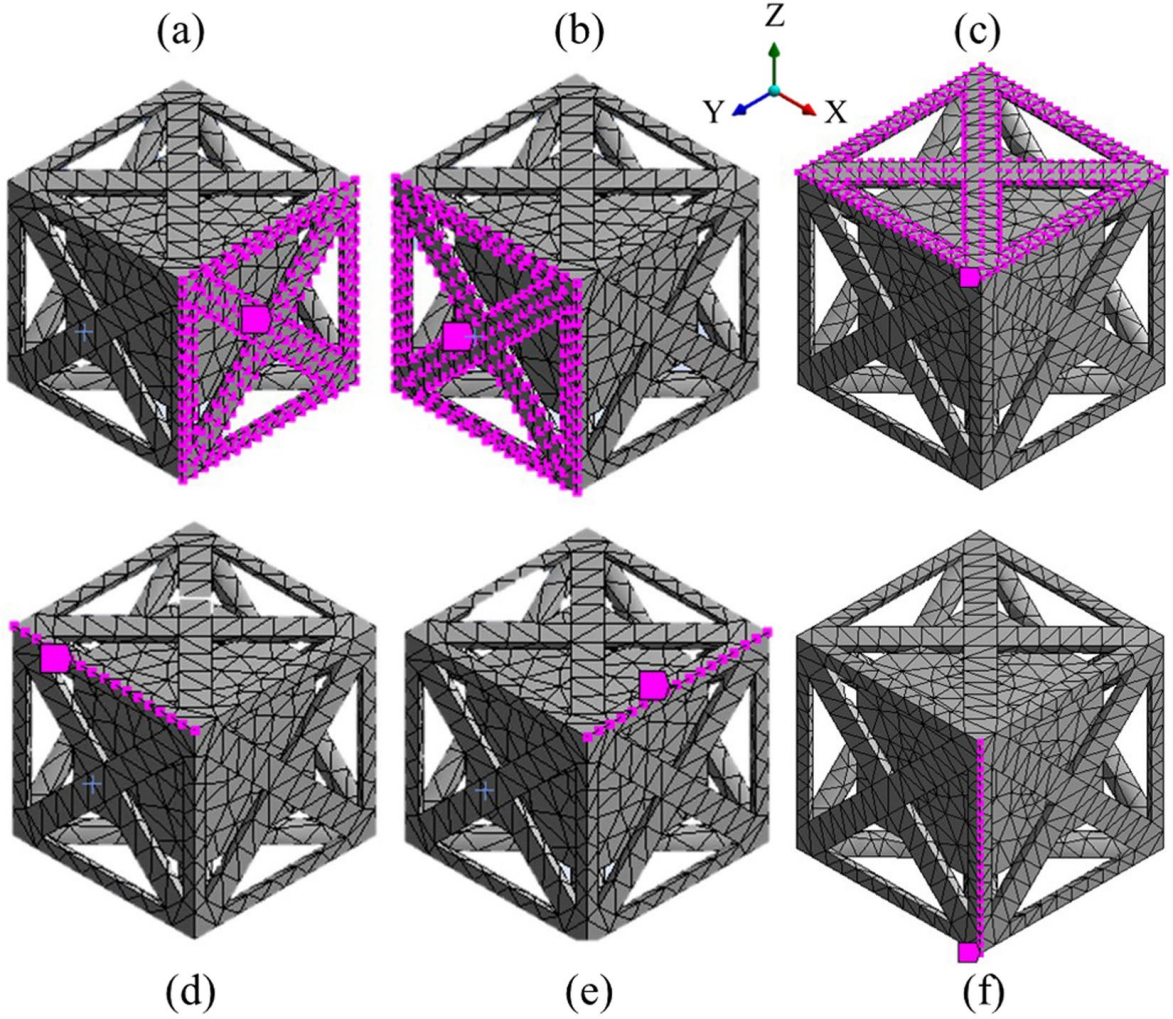
Selection of the segmentation of the unit cell of the metamaterial into DOF as used for modelling the periodicity of the unit cell. The magenta points represent the (**a**) front nodes, (**b**) left nodes, (**c**) top nodes, (**d**) top-left nodes, (**e**) top-front nodes, and (**f**) front-left nodes.

The proposed scheme for computing the dispersion curves used a combination of FE modelling and periodic structure theory. The metamaterials were modelled using FE modelling which allows for accurate representation of the geometrically complex metamaterials. The complete mass and stiffness matrices of the designs, ***K*** and, ***M*** respectively, were extracted. The Bloch theorem^59^, which governs the periodic displacement and forcing conditions was employed. The periodic structure theory assumed an infinite 3D spatial periodicity of the unit cell^60,61^. Figure 4 is a schema of the segmentation of the unit cell of the metamaterial into sets of DOF, which were used for modelling the periodicity of the unit cell.

The nodal displacement matrices *q* were arranged in the following sequence to allow for the 3D spatial periodicity of the unit cell

1 $${\boldsymbol{q}}={[{{\boldsymbol{q}}}_{IN}{{\boldsymbol{q}}}_{F}{{\boldsymbol{q}}}_{S}{{\boldsymbol{q}}}_{B}{{\boldsymbol{q}}}_{T}{{\boldsymbol{q}}}_{L}{{\boldsymbol{q}}}_{R}{{\boldsymbol{q}}}_{FB}{{\boldsymbol{q}}}_{FT}{{\boldsymbol{q}}}_{SB}{{\boldsymbol{q}}}_{ST}{{\boldsymbol{q}}}_{FL}{{\boldsymbol{q}}}_{FR}{{\boldsymbol{q}}}_{SL}{{\boldsymbol{q}}}_{SR}{{\boldsymbol{q}}}_{BL}{{\boldsymbol{q}}}_{BR}{{\boldsymbol{q}}}_{TL}{{\boldsymbol{q}}}_{TR}]}^{{\rm{T}}},$$

where the subscripts *IN*, *L*, R, *T*, *B*, *F*, and *S* indicate the DOF of the nodes existing at the inside, left, right, top, bottom, front, and back of the unit cell as illustrated in Figure 4. A transformation matrix *R* was considered to project the nodal displacement matrices as follows

2 $${\boldsymbol{q}}={\boldsymbol{R}}\tilde{{\boldsymbol{q}}},$$

where

3 $${\boldsymbol{R}}=[\begin{array}{ccccccc}{\boldsymbol{I}} & {\bf{0}} & {\bf{0}} & {\bf{0}} & {\bf{0}} & {\bf{0}} & {\bf{0}}\\ {\bf{0}} & {\boldsymbol{I}} & {\bf{0}} & {\bf{0}} & {\bf{0}} & {\bf{0}} & {\bf{0}}\\ {\bf{0}} & {\boldsymbol{I}}{e}^{-i{k}_{y}} & {\bf{0}} & {\bf{0}} & {\bf{0}} & {\bf{0}} & {\bf{0}}\\ {\bf{0}} & {\bf{0}} & {\boldsymbol{I}} & {\bf{0}} & {\bf{0}} & {\bf{0}} & {\bf{0}}\\ {\bf{0}} & {\bf{0}} & {\boldsymbol{I}}{e}^{-i{k}_{z}} & {\bf{0}} & {\bf{0}} & {\bf{0}} & {\bf{0}}\\ {\bf{0}} & {\bf{0}} & {\bf{0}} & {\boldsymbol{I}} & {\bf{0}} & {\bf{0}} & {\bf{0}}\\ {\bf{0}} & {\bf{0}} & {\bf{0}} & {\boldsymbol{I}}{e}^{-i{k}_{x}} & {\bf{0}} & {\bf{0}} & {\bf{0}}\\ {\bf{0}} & {\bf{0}} & {\bf{0}} & {\bf{0}} & {\boldsymbol{I}} & {\bf{0}} & {\bf{0}}\\ {\bf{0}} & {\bf{0}} & {\bf{0}} & {\bf{0}} & {\boldsymbol{I}}{e}^{-i{k}_{z}} & {\bf{0}} & {\bf{0}}\\ {\bf{0}} & {\bf{0}} & {\bf{0}} & {\bf{0}} & {\boldsymbol{I}}{e}^{-i{k}_{y}} & {\bf{0}} & {\bf{0}}\\ {\bf{0}} & {\bf{0}} & {\bf{0}} & {\bf{0}} & {\boldsymbol{I}}{e}^{-i{k}_{y}}{e}^{-i{k}_{z}} & {\bf{0}} & {\bf{0}}\\ {\bf{0}} & {\bf{0}} & {\bf{0}} & {\bf{0}} & {\bf{0}} & {\boldsymbol{I}} & {\bf{0}}\\ {\bf{0}} & {\bf{0}} & {\bf{0}} & {\bf{0}} & {\bf{0}} & {\boldsymbol{I}}{e}^{-i{k}_{x}} & {\bf{0}}\\ {\bf{0}} & {\bf{0}} & {\bf{0}} & {\bf{0}} & {\bf{0}} & {\boldsymbol{I}}{e}^{-i{k}_{y}} & {\bf{0}}\\ {\bf{0}} & {\bf{0}} & {\bf{0}} & {\bf{0}} & {\bf{0}} & {\boldsymbol{I}}{e}^{-i{k}_{x}}{e}^{-i{k}_{y}} & {\bf{0}}\\ {\bf{0}} & {\bf{0}} & {\bf{0}} & {\bf{0}} & {\bf{0}} & {\bf{0}} & {\boldsymbol{I}}\\ {\bf{0}} & {\bf{0}} & {\bf{0}} & {\bf{0}} & {\bf{0}} & {\bf{0}} & {\boldsymbol{I}}{e}^{-i{k}_{x}}\\ {\bf{0}} & {\bf{0}} & {\bf{0}} & {\bf{0}} & {\bf{0}} & {\bf{0}} & {\boldsymbol{I}}{e}^{-i{k}_{z}}\\ {\bf{0}} & {\bf{0}} & {\bf{0}} & {\bf{0}} & {\bf{0}} & {\bf{0}} & {\boldsymbol{I}}{e}^{-i{k}_{x}}{e}^{-i{k}_{z}}\end{array}],\,\mathrm{and}\,\tilde{{\boldsymbol{q}}}=[\begin{array}{c}\begin{array}{c}{{\boldsymbol{q}}}_{IN}\\ {{\boldsymbol{q}}}_{F}\end{array}\\ {{\boldsymbol{q}}}_{B}\\ \begin{array}{c}{{\boldsymbol{q}}}_{L}\\ {{\boldsymbol{q}}}_{FB}\\ \begin{array}{c}{{\boldsymbol{q}}}_{FL}\\ {{\boldsymbol{q}}}_{BL}\end{array}\end{array}\end{array}],$$

where *k* is the wavenumber for the waves propagating in *x*-, *y*- and *z*- directions within the considered regions of the IBZ. Subsequently, the projected stiffness and mass matrices of the reduced sets of DOF, $\bar{{\boldsymbol{K}}}$ and $\bar{{\boldsymbol{M}}}$, were computed as

4 $$\bar{{\boldsymbol{K}}}={\boldsymbol{R}}{\boldsymbol{^{\prime} }}{\boldsymbol{KR}},\,{\rm{and}}\,\bar{{\boldsymbol{M}}}={\boldsymbol{R}}{\boldsymbol{^{\prime} }}{\boldsymbol{MR}}{\boldsymbol{,}}$$

Assuming no external excitation under Bloch-Floquet^59^ boundary conditions, the following eigenvalue problem was derived in the wave domain

5 $$(\bar{{\boldsymbol{K}}}-{\omega }^{2}\bar{{\boldsymbol{M}}}){\boldsymbol{\phi }}=0,$$

where *ω* is the angular frequency and ***φ*** is the eigenvector. Eq. 5 provided the wave propagation characteristics of the metamaterials in 3D space. By substituting a set of presumed wavenumbers in a given direction, the derived eigenvectors ***φ*** provided the deformation of the unit cell under the passage of each wave type at an angular frequency *ω*. To obtain normalised frequencies, the frequency eigenvalues of Eq. 5 were normalised to the unit cell size *C* and the speed of longitudinal waves in the lattice material *v*, which was calculated as the square root of the quotient of the elastic modulus and material density. A complete description of each passing wave, including *x*-, *y*- and *z*-directional wavenumbers and wave shapes, at a certain frequency range is acquired with modulo 2π. When modelling the dispersion curves of the metamaterial used in this work, suitable 3D translation of all solid features and voids within the unit cell is obtained when the design is approximated as a simple cube, thus, allowing for the use of the IBZ of simple cubic lattice for modelling the dispersion curves. Such approximation is known to provide accurate dispersion relations as can be seen elsewhere^62–64^. The computation did not include damping, though it should be noted that structural damping can be directly introduced to Eq. 4 by including an imaginary part of the $\,\bar{{\boldsymbol{K}}}$ matrix^65^. Alternatively, if full viscous damping properties are to be considered, then dedicated eigenvalue problem solvers can be employed^59^.

### Additive manufacturing technology employed

Internally resonating metamaterial samples were fabricated on a laser powder bed fusion (LPBF) system using PA12 polymer material. The material properties for PA12 can be found in Table 1. The LPBF system used a 21 W laser of scan speed and hatch spacing of 2500 mm⋅s^−1^ and 0.25 mm, respectively. The nominal spot size of the laser was 0.3 mm and the layer thickness was 0.1 mm. PA12 powder was used to fill the powder bed of dimensions 1320 mm × 1067 mm × 2204 mm at a temperature of 173 °C. Geometrical features of sizes below 0.8 mm are usually manufactured with considerable losses in mechanical properties, due to the existence of unsolidified powder within the manufactured features^57^. To ensure that all geometrical features were manufactured in agreement with the specified design, the size of the narrowest metamaterial feature was designed to be 1 mm^57^.

**Table 1:** Material properties of PA12^69^.

Material property	Value
Young’s modulus	1.5 × 10^3^ MPa
Density	950 kg⋅m^−3^

### Experimental measurements on vibration transmissibility

The metamaterial sample was suspended using piano strings to approximate free-free boundary conditions. The approach taken to suspend the metamaterial, similar to the approach taken by Zhang *et al*.^48^ and Chen *et al*.^66^, supports the metamaterial uniformly. An alternative approach, which can also be used for approximation of free-free boundary conditions, can be found in the work of D’Alessandro *et al*.^47^. The metamaterial was adhesively affixed from one side to a connector which was, in turn, bolted to an acceleration sensor. The acceleration sensor was linked to the armature of the shaker (Modal Shop Shaker 2060E)^67^ through a stinger. The stinger is a 1.5 mm rod which connects to the acceleration sensor, and decouples cross-axis force inputs, thus, minimising errors during measurements^68^. As part of the experimental setup, the beam of a laser vibrometer was projected perpendicularly to the opposite surface of the metamaterial to take longitudinal acceleration measurements. The transverse acceleration measurements were taken by projecting the beam of the laser vibrometer perpendicularly to the side surfaces of the metamaterial. The laser vibrometer was set to measure the structural response in the longitudinal and transverse directions from a normalised frequency of 0 to 0.15. The acceleration data within the tested frequency range were also obtained through the acceleration sensor. The combination of the measurements of both the laser vibrometer and the acceleration sensor provided the transmissibility of the specimen. Figure 3c is a representative photograph of the experimental setup. All measurements were taken with a normalised frequency resolution of less than 3.7 × 10^−5^ and were complexly averaged, considering both the phase and the magnitude of the measurements, over 100 spectral sweeps.
